# Qizhu Yuling prescription in the prevention of postoperative metastasis and recurrence of esophagus cancer: study protocol for a randomized, double-blind, placebo-controlled, multicenter clinical trial

**DOI:** 10.3389/fonc.2025.1478390

**Published:** 2025-03-26

**Authors:** Miao Kong, Bowen Xu, Guanghui Zhu, Xinmiao Wang, Ziyu Kuang, Qianhui Sun, Kexin Liu, Zilin Wang, Ying Zhang, Jie Li

**Affiliations:** ^1^ Department of Oncology, Guang’anmen Hospital, China Academy of Chinese Medical Sciences, Beijing, China; ^2^ Department of TCM/Integrative Medicine, Hunan Cancer Hospital, Changsha, China; ^3^ Graduate School, Beijing University of Chinese Medicine, Beijing, China; ^4^ Department of Oncology, The First Affiliated Hospital of Zhejiang Chinese Medical University (Zhejiang Provincial Hospital of Traditional Chinese Medicine), Hangzhou, China

**Keywords:** Qizhu Yuling prescription, esophageal cancer, traditional Chinese medicine, randomized controlled trial, disease-free survival, protocol

## Abstract

**Background:**

Esophageal cancer (EC) is a malignant tumor with a high recurrence and metastasis rate and poor prognosis. In 2024, China ranked first in the world in terms of new EC cases and deaths. Surgery is the main treatment method for EC, but the clinical difficulty is how to prevent recurrence and metastasis after surgery. Traditional Chinese medicine as a complementary therapy has played an important role in this regard. Preclinical studies have confirmed that Qizhu Yuling Prescription (QZYLP) has anticancer effects, reduces treatment side effects, and improves quality of life, except for the lack of long-term prognostic results. Therefore, this study aims to investigate whether QZYLP can reduce the recurrence and metastasis rates of EC after surgery, improve disease-free survival (DFS), prolong overall survival, and observe the safety of the drug.

**Methods:**

This study is a multicenter, randomized, double-blind, placebo-controlled clinical trial. It seeks to enroll 310 patients from 10 hospitals who have completed adjuvant therapy following R0 surgery for esophageal squamous cell carcinoma without recurrent metastasis. Using a center-randomized design, participants will be assigned to the control group (n=155, receiving placebo treatment) or experimental group (n=155, receiving QZYLP granules treatment). Treatment will last for 6 months, with follow-up every 3 months after the final treatment or endpoint event, continuing for up to 3 years postoperatively. The primary outcome measured is DFS at 1 year postoperatively. Secondary outcomes included indicators related to prognosis, fat distribution, peripheral blood inflammation, tumor markers, and quality of life scales.

**Discussion:**

This study aims to further clarify the efficacy and safety of QZYLP in preventing postoperative recurrence and metastasis of EC, and to explore the mechanism of action. The results of this study will provide high-quality evidence for the participation of TCM in the comprehensive treatment program of EC, and improve the precise diagnosis and treatment system of TCM in EC.

**Clinical trial registration:**

ClinicalTrials.gov, identifier NCT05626309.

## 1 Introduction

Esophageal cancer (EC) is one of the deadliest cancers globally. According to the Global Cancer Statistics 2022, the number of new EC cases worldwide is 511,054, ranking fourth among digestive system cancers (eleventh among all malignancies). The number of deaths was 445,391, ranking fifth among digestive system cancers (seventh among all malignancies). China ranked first in the world in terms of new EC cases and deaths, accounting for 43.8% of new cases and 42.1% of deaths (1). Chinese age-standardized incidence rate and mortality rate of EC were 8.32/10^5^ (5.0/10^5^ worldwide) and 6.68/10^5^ (4.3/10^5^ worldwide) in 2022 respectively (2). EC is a highly aggressive malignant tumor with a poor overall prognosis. In China, the surgical resection rate for EC currently ranges from 58%~92% (3). In the management of operable esophageal adenocarcinoma and certain squamous cell carcinoma cases, the established treatment paradigm incorporates induction therapy (chemotherapy with or without radiation), surgery, and postoperative adjuvant therapy. However, the total recurrence rate after radical resection is as high as 27–52.4% (4), significantly influencing patient prognosis. The *2023 NCCN Clinical Practice Guidelines in Oncology* (5) emphasize that the main focus of EC and esophagogastric tumor treatment is the management of recurrence and metastasis. Currently, the strategies to prevent recurrence and metastasis of EC after operation mainly include radiotherapy, chemotherapy, and immunotherapy. However, these treatments often face challenges such as drug resistance and toxic side effects (6–8). Moreover, the recurrence and metastasis rates are much higher during the window period after adjuvant therapy (4). Therefore, exploring new treatment strategies in this window period is crucial as standard treatments remain insufficient (9).

Throughout thousands of years of development, Traditional Chinese Medicine (TCM) has consistently been used to emphasize and advocate for preventive treatment. Increasing evidence indicates that TCM can effectively mitigate the recurrence and metastasis of malignancies (10, 11). A recent national multicenter prospective cohort study has demonstrated that the integration of TCM in the treatment of patients with post-operative stage II-IIIA non-small cell lung cancer (NSCLC) is associated with a reduction in recurrence rates by approximately 39% (12). Furthermore, a meta-analysis of randomized controlled trials has confirmed the efficacy and safety of compound kushen injection for adults with esophageal cancer (13). The Department of Oncology, Guang’anmen Hospital, China Academy of Chinese Medical Sciences (CACMS), has long been dedicated to the treatment of EC. Based on clinical experience, they have developed a Chinese herbal compound formula known as Qizhu Yuling Prescription (QZYLP). The formula consists of *Astragali Radix, Codonopsis Radix, Rhizoma Atractylodis Macrocephalae, Rhizome Curcumae, Clematidis Radix et Rhizoma, Stalactite*, and Chinese Sage Herb. This compound formula has been declared an invention patent in the People’s Republic of China, with Application No. 202210873081.0. A previous study employed QZYLP to intervene in patients with EC after postoperative radiotherapy and chemotherapy, including 55 prospective cases and 123 retrospective cases. The results showed that the median Disease-free Survival (DFS) was 43 and 31 months, respectively (14), which were both prolonged compared with similar periods in modern medical studies (15). Furthermore, the study demonstrated that QZYLP can alleviate symptoms such as fatigue, shortness of breath, and dysphagia, and it can improve quality of life, with no serious adverse effects observed. Through thorough preliminary research, QZYLP was found to exhibit anticancer effects, reduce treatment side effects, and enhance quality of life. However, there is a lack of national multicentre evidence-based support for the long-term efficacy of QZYLP in post-EC patients.

Therefore, this study aims to investigate the long-term efficacy and safety of QZYLP for preventing recurrent metastasis in postoperative EC through a large-scale, multicenter, high-quality, randomized controlled trial (RCT).

## 2 Materials and methods

### 2.1 Study design

**Figure 1:**
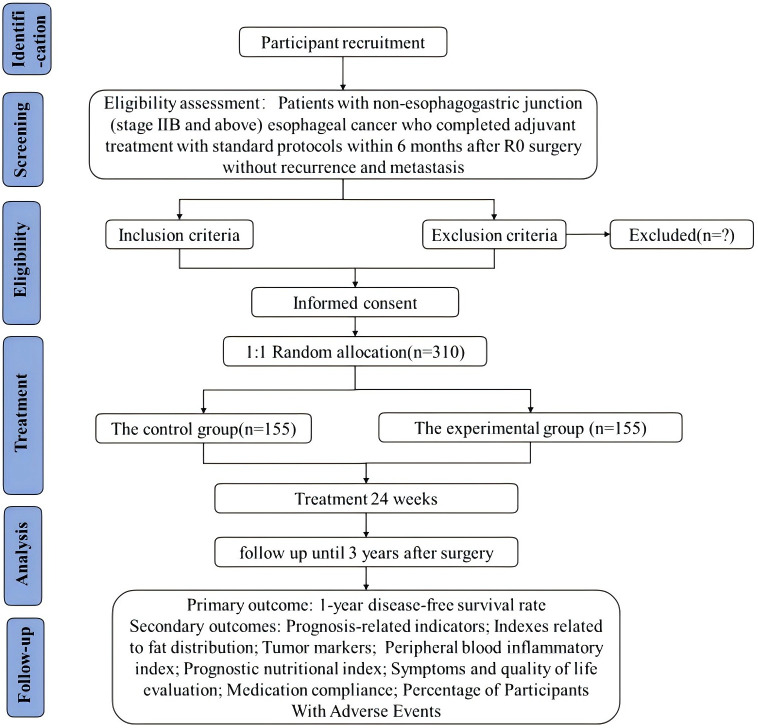
Study flow chart.

**Figure 2:**
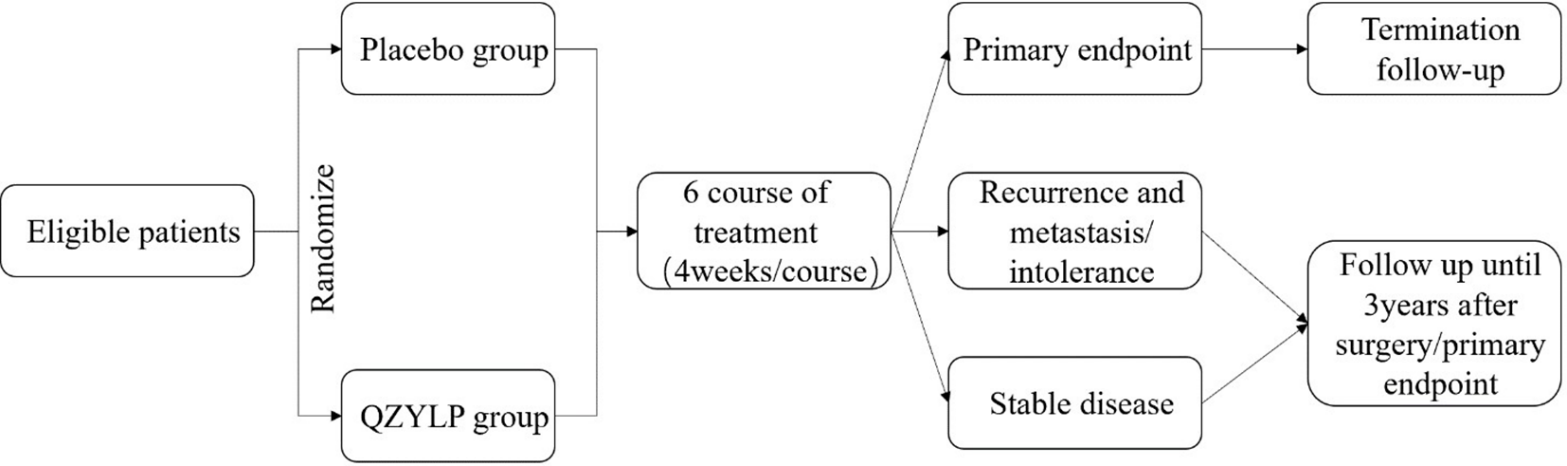
Intervention and follow-up flow chart.

This study is a multicenter, randomized, double-blind, placebo, parallel-controlled clinical trial conducted according to the protocol approved by the Medical Ethics Committee of Guang’anmen Hospital (2022-200-KY-01). It was registered with Clinical Trials (https://clinicaltrials.gov/) under the number NCT05626309. A randomized design is implemented using a centralized randomization system to conceal protocol allocation. The control group receives a placebo, while the experimental group is treated with QZYLP granules. Both groups also undergo conventional symptomatic therapy. Treatment duration spans 6 months, with follow-up continuing for 3 years post-surgery. After completing the final treatment or reaching the endpoint event, all patients entered a post-treatment follow-up period with evaluation every 3 months. The primary endpoint is the 1-year DFS rate after surgery, which is used to measure the proportion of patients who do not experience recurrence, metastasis, or death (from any cause) within 1 year following surgery. **Figure 1** shows the timeline for enrollment, intervention, and evaluation, while **Figure 2** shows the timeline for intervention and follow-up. **Table 1** depicts the study flowchart.

**Table 1:** Flowchart of treatment phases.

Project		Lead-in period	Intervention period				Follow-up period
Timepoint		1	End of the first course of treatment	End of the second course of treatment	End of the third course of treatment	End of the N course of treatment	N+X
Week		-W1-W0	W4-W5	W8-W9	W12-W13	W4N-W4N+1	Every 3 months
Inclusion/exclusion criteria		✓					
Informed consent		✓					
Medical history							
Demographic data		✓					
Diagnosis		✓					
Therapeutic observation							
Vital signs		✓	✓	✓	✓	✓	
Clinical examination		✓	✓	✓	✓	✓	✓
Physical condition score		✓	✓	✓	✓	✓	✓
Clinical symptoms		✓	✓	✓	✓	✓	
Description of tongue and pulse		✓	✓	✓	✓	✓	
TCM syndrome		✓	✓	✓	✓	✓	
Imaging Examination	PET-CT/CT	✓			✓	✓	✓
	MRI、B-ultrasound	*			*	*	*
	Bone scanning	*			*	*	*
	Gastroscope	*			*		*
Tumor marker		✓	✓	✓	✓	✓	✓
Indexes related to fat distribution		✓	✓	✓	✓	✓	✓
MDASI-TCM		✓	✓	✓	✓	✓	
QLQ-QES18		✓	✓	✓	✓	✓	
Safety observation							
Routine blood tests		✓	✓	✓	✓	✓	✓
Liver/kidney function		✓	✓	✓	✓	✓	✓
Routine urine/stool tests		*	*	*	*	*	*
ECG		✓	✓	✓	✓	✓	✓
CTCAE			✓	✓	✓	✓	
Other work							
Drug combination			✓	✓	✓	✓	
Efficacy evaluation			✓	✓	✓	✓	✓
Treatment							✓

**Table 1** The schedule of participants. *Indicates: This examination is optional. For imaging examinations, the same examination method should be used for screening of the same site and for each review after enrollment, and the examination should be reviewed every 3 sessions during the treatment phase and every 3 months during the follow-up phase. Head, chest, and abdominal examinations should be selected as appropriate. If CT/MRI examination has been done for the same site, abdominal X-ray examination and ultrasound examination are not necessary. Bone scanning examination is selected according to the patient’s condition. TCM, Traditional Chinese Medicine. PET-CT, Positron Emission Tomography Computed Tomography. CT, Computed Tomography. ECG, Electrocardiogram. MRI, Magnetic Resonance Imaging. MDASI-TCM, M.D.Anderson Symptom Traditional Chinese Medicine. QLQ-QES18, Quality of Life Questionnaire of Oesophageal-Specific Module 18. CTCAE, Common Terminology Criteria for Adverse Events. ✓ Indicates: This work needs to be performed.

### 2.2 Participant screening and selection

The cases for this study were sourced from a real-world postoperative EC registry platform. This study pre-integrates advantageous units from Chinese medicine EC research (Additional file 1. Attended hospitals), including oncology advantageous departments in general hospitals and specialized gastrointestinal oncology advantageous departments. The goal was to standardize and collect clinical information on postoperative patients with EC and build a postoperative EC registry platform. This platform will support evidence-based studies by providing a comprehensive case database.

Based on the characteristics of the primary target population of QZYLP identified in the previous study, postoperative patients with EC from the registry platform were selected according to the following criteria:

Diagnostic criteria for EC were based on the *Guidelines of the Chinese Society of Clinical Oncology (CSCO) for Esophageal cancer 2021* (16). Pathological staging criteria follow the *World Health Organization (WHO) Classification of Tumors of the Digestive System, 5^th^ edition* (17). TNM staging criteria refer to the *Cancer of the Esophagus and Esophagogastric Junction: An Eighth Edition Staging Primer* (18). For patients who met the diagnostic criteria, the inclusion and exclusion criteria (listed below) were reassessed. Patients will not be recruited if they do not meet any of the inclusion criteria or meet any of the exclusion criteria.

### 2.3 Inclusion criteria

1. EC without esophagogastric junction pT_1-4a_N_+_M_0_ (stage IIB-IVA) that met the diagnostic criteria without recurrence or distant metastasis;
2. Patients who completed adjuvant therapy (including adjuvant radiotherapy, chemotherapy, chemotherapy + radiotherapy) within 6 months after R0 resection for EC.
3. ECOG score 0–2.
4. 18–75 years old.
5. Expected survival ≥ 3 months.
6. Participants who voluntarily signed informed consent.

### 2.4 Exclusion criteria

1. Presence of primary tumor at other sites.
2. Patients with severe primary diseases affecting the heart, cerebral vessels, liver, kidney, or hematopoietic system.
3. Patients with mental illness and mental and language impairments.
4. Participation in other clinical trials within the past 3 months.
5. Patients with known hypersensitivity or intolerance to study drug.

### 2.5 Drop out criteria

1. Patients who are unable to adhere to the treatment owing to unforeseen events during the study.
2. Participants who voluntarily request to withdraw from the study.
3. Participants with poor adherence to the study protocol as determined by the investigator.
4. Pregnancy, death, or loss of contact with the participant.

Following termination of treatment or withdrawal from the study, they entered a follow-up period. They received periodic follow-ups (every 3 months, with telephone follow-up accepted) to assess survival status, extending for at least 3 years post-operation.

### 2.6 Randomization

Central and compartmentalized randomizations were conducted using the Interactive Web Response System (IWRS) to ensure allocation scheme concealment. The test and control groups were randomized in a 1:1 ratio through the central randomization grouping method. Using R software (V3.3.3), three cycles of random statements were employed to generate random sequences, creating a list of random codes corresponding to serial numbers 001–310. Researchers accessed the central randomization system website, inputting necessary details (such as center number, patient initials, and contact phone number), and the system generated group assignments and corresponding random numbers for the cases. The protocol was followed based on the assigned groupings. If a participant inadvertently used the wrong group of drugs, no corrections were made, and the original drug treatment regimen was maintained. Details of the drug treatment were recorded in the case report form.

### 2.7 Blinding

This is a double-blind study, ensuring that the investigator and participants remained unaware of the study details. Statisticians who are not involved in the clinical trial conducted the blinding process. The random code table, created by the research unit, was securely sealed and stored separately. The study drugs were manufactured, packaged, and supplied by Jiangyin Tianjiang Pharmaceutical Co., with the randomization code serving as the unique identification code for participants. Supervisors and outcome evaluators remained blinded throughout the study.

### 2.8 Emergency blindness breaking

For this trial, a dedicated “emergency letter” system was established, where each drug was assigned a specific emergency letter. The actual treatment group (test/control) corresponding to each drug was indicated in a specific area of the emergency letter, covered by a disposable, fragile coating. Emergency unblinding was only performed in the event of a participant emergency where managing the situation required clarification of the medication used. In the event of an emergency requiring unblinding, the investigator will consult with the center in charge. Upon receiving signed approval, the investigator can open the emergency blinding letter and record the event. The unit in charge will be notified within 24 h after the unblinding.

### 2.9 Sample size calculation

This study is a RCT. The test group was administered QZYLP Granules, while the control group was placed on a placebo. The primary outcome measure was the postoperative DFS of patients. The study was designed as a superiority trial with a 1:1 ratio between the test and control groups. According to the literature, the mDFS for patients with lymph node-positive esophageal squamous carcinoma after surgery with adjuvant therapy is 19.3 months (19). Assuming that the DFS of the test group was 12 months longer than that of the control group, with α=0.05 and 1-β=0.80, the expected time to enroll all participants was 24 months, and the total duration of the clinical trial was expected to be 36 months, with a consistent enrollment rate of the study participants. Using PASS 11 software, it was calculated that 140 cases were needed in each of the test and control groups. Considering a 10% dropout rate, 155 participants were enrolled in the test and control groups, resulting in a final number of 310 participants. The following formula was used (20). Here δ=π_1_-π_2_.


$$n={\left(\frac{u_{\alpha }+u_{\beta }}{\delta }\right)}^{2}[\pi_{1}(1-\pi_{1})+\pi_{2}(1-\pi_{2})]$$

### 2.10 Interventions

The experimental group was administered QZYLP granules. Patients were administered two small packets in the morning and two small packets in the evening. The medication was dissolved in hot water before administration. Each course of treatment lasted 4 weeks, with six courses in total. The patients will be followed up for up to 3 years after their EC surgery.

The control group was administered a placebo. The placebo was designed to be identical to the experimental drug in terms of the outer packaging, color, shape, and taste. It was prepared from maltodextrin and food coloring. The raw materials of maltodextrin conformed to the relevant provisions of excipients - maltodextrin in the *Chinese Pharmacopoeia 2015 edition, Part Four* (21). The food coloring included caramel coloring, egg yolk coloring, and milk chocolate brown pigment, all of which were edible-grade ingredients. The dosing method was comparable to that of the experimental group.

During the experimental treatment, drugs for managing myelosuppression, nausea and vomiting, diarrhea, abnormal liver and kidney functions, infections, and other symptomatic treatments were allowed concurrently. However, the specific symptoms and the combined use of these drugs were accurately recorded. The following were prohibited: (1) Other antitumor therapies (including chemotherapy, immunotherapy, molecular targeted therapy, and radiotherapy) (2) Chinese herbal decoctions, Chinese herbal injections, and Chinese patent medicine with antitumor effects.

### 2.11 Outcome measures

#### 2.11.1 Primary outcome

1-year DFS rate: Refers to the proportion of patients who did not experience recurrence, metastasis, or death (from any cause) within 1 year following surgery. Although OS is the gold standard for evaluating malignant tumor treatment, given the strong correlation between DFS and OS and to control of time costs, the 1-year DFS rate was selected as the primary endpoint to explore long-term efficacy (22).

#### 2.11.2 Secondary outcomes

(1) Prognosis-related indicators:

1. DFS: Time from randomization to tumor progression or death (from any cause).
2. Overall survival (OS): Time from randomization to death (from any cause).
3. Cumulative annual recurrence and metastasis rate for 1–3 years: Proportion of patients experiencing recurrence and metastasis from the day of surgery to 1, 2, and 3 years.
4. Cumulative annual survival rate for 1–3 years: Proportion of patients surviving from the day of surgery to 1, 2, and 3 years.

(2) Indexes associated with fat distribution:

1. Total, Visceral, and Subcutaneous Fat Areas: These measure fat areas on cross-sectional images obtained through plain CT scanning.
2. Visceral Adiposity Index: A new assessment of visceral fat based on waist circumference (WC), body mass index (BMI), triglyceride (TG), high-density lipoprotein (HDL), and visceral adiposity index (VAI). Male VAI = during [WC/(39.68 + 1.88 × BMI)] × (TG/1.03) × (1.31/HDL). Female VAI = during [WC/(36.58 + 1.89 × BMI)] × (TG/0.81) × (1.52/HDL).

(3) Tumor markers: squamous cell carcinoma antigen (SCC), carcinoembryonic antigen (CEA), carbohydrate antigen 19-9 CA199), and cytokeratin-19-fragment (CYFR21-1).
(4) Peripheral blood inflammatory index: lymphocyte-to-monocyte ratio (LMR); lymphocyte-to-neutrophil ratio (LNR).
(5) Prognostic nutritional index: Serum albumin level (g/L) + 5 × total number of peripheral blood lymphocytes (×10^9/L).
(6) Symptoms and quality of life evaluation:

1. Quality of life of the patient: This score was assessed using the Quality of Life Questionnaire Esophageal-Specific Module 18(QLQ-QES18). Each of the 18 questions is scored from 1 to 4, with a total score ranging from 18 to 72. A higher score indicates poorer quality of life.
2. Evaluation of the symptoms of the patients: This score was measured using the M. D. Anderson Symptom Traditional Chinese Medicine (MDASI-TCM). Each of the 26 questions was rated on a scale from 0 to 10, resulting in a total score ranging from 0 to 260. A higher score indicated more severe symptoms.

(7) Medication compliance: The number of cases and percentages were calculated for <80, 80–120, and >120% compliance.
(8) Percentage of Participants with Adverse Events: Percentage of participants experiencing adverse events in different study arms.

#### 2.11.3 Safety outcomes

Routine blood/urine/stool tests, liver and kidney function, and electrocardiogram (ECG), among others.

### 2.12 Adverse events observation and recording

Adverse event monitoring in this study followed the Common Terminology Criteria for Adverse Events developed by the U.S. Department of Health and Human Services. All adverse events experienced by participants during the study were documented in the Case Report Form (CRF). Adverse events were identified through self-reports by participants, test results, and retrospective review of medication co-administration. Upon the occurrence of an adverse event, the investigator assessed whether the participant should continue in the study based on the severity. Cases where participants discontinued the trial owing to serious adverse events were followed up, and their outcomes were documented.

### 2.13 Data collection and management

#### 2.13.1 Data collection

Raw data will be obtained from the medical records of the participants, test and examination reports issued by medical institutions at all levels, and patient interview scale data. Investigators at each sub-center will collect and enter this information into paper CRFs. The study data will be managed using the Jdhhealth Multi-center research collaboration platform, an Electronic Data Capture System (EDC) hosted by Beijing Jiudu Jiade Technology Co. Jdhhealth is a secure web-based platform for data collection, tracking, verification, and export. It supports multi-user access and prevents unauthorized entry through established security protocols.

#### 2.13.2 Data management

Two trained researchers will perform double data entry consecutively. The EDC system will be used to compare and verify the double data entry, after which the researchers conduct final data modification and validation. The EDC system will keep a log of data changes. Participants will be identified in the system by code rather than by name. Once data quality control is finalized, the database will be locked. The researchers will assume data monitoring responsibilities.

### 2.14 Statistical analysis

Statistical software: SAS 9.4 statistical software will be utilized to analyze effective indicators using the Full Analysis Set and Per Protocol Set analyses. A safety data set analysis will be performed for adverse reactions.

Statistical methods: All statistical tests will be two-sided, with *P*≤ 0.05 considered statistically significant for the differences tested. Data will be analyzed for outliers, which are professionally evaluated to determine appropriate trade-offs. Missing values will also be professionally analyzed to determine whether to classify them as dropouts or data transfers. The proportion of missing cases should not exceed 10%; otherwise, they should be analyzed and explained. Measurement data will be described using mean, standard deviation, median, minimum, and maximum values. Count data will be described using frequency and percentage. The *T*-test and rank-sum test, among others, will be employed to analyze measurement data, while the chi-square test and Ridit analysis will be used for count data. Survival data will be analyzed using the Kaplan–Meier method, Wilcoxon rank-sum test, and log-rank test. For multifactorial survival analysis, the Cox proportional risk regression model will be used. To reveal whether there are differences in effects between different feature groups, subgroup analyses will be performed on subgroups for sex, age, occupation, health status (smoking, alcohol using, hypertension, diabetes, etc), type of operation, and adjuvant therapies.

### 2.15 Quality control

To protect the rights and interests of the participants, the investigator will comply with the *The International Council for Harmonization (ICH) ICH-E6 Good Clinical Practice (GCP)* (23), ensuring the scientific validity, reliability, accuracy, and completeness of the study.

#### 2.15.1 Quality control

The principal investigator (PI) is responsible for the overall quality control of the study, which is specifically implemented and executed by individual investigators and other participants. This included: ① Regularly verifying the operation of the postoperative EC case registry platform. ② Ensuring all investigators adhering strictly to standard operating procedures and trial protocols. ③ Recording clinical data in a timely, direct, accurate, and clear manner, including signatures and dates. ④ Frequently self-checking the accuracy and completeness of data records and using prescribed methods to correct errors. (5) Using validated and reliable statistical software for data analysis. (6) Implementing effective quality control measures for data entry, such as double entry.

#### 2.15.2 Monitor

Individuals are appointed by the PI to oversee the entire clinical trial. Their responsibilities included the following: ① Before the trial: selecting investigators, assisting in the development of trial documents, preparing trial materials, and organizing investigator meetings; ② During the trial: conducting regular monitoring, managing and supplying trial materials, monitoring trial progress and confirming informed consent is obtained, ensuring investigators comply with the trial protocol, verifying original information and regularly accessing trial documents, managing the trial drug and storing the blinding code, recording and reporting adverse events, and timely submission of monitoring reports to the PI; ③ At the end of the trial: conducting a final visit, recovery of trial materials, preserving trial data, and assisting the investigator in reporting trial results to the PI.

#### 2.15.3 Audit

Clinical trial PIs should engage a quality assurance department or a third party to conduct audits. The auditor will be independent of the clinical trial. The main responsibilities include reviewing the original trail information and reports, conducting internal and external audits, maintaining relevant documents, and offering guidance and training to investigators and those monitoring the trails.

### 2.16 Ethical approval and confidentiality

The study was approved by the Medical Ethics Committee of Guang’anmen Hospital, CACMS (2022-200-KY-01), and will be conducted under its supervision. All participants will provide written informed consent before enrollment to ensure voluntary participation. Laboratory samples, reports, and data collection will be identified by code instead of the names of participants. All study-related paper materials will be securely stored in locked file cabinets to protect the confidentiality of the personal medical information of the participants.

## 3 Discussion

Currently, EC has caused a huge medical burden, and the OS benefit is still limited in spite of the advances in surgical techniques and optimization of radiotherapy regimens (24). The reason is that metastasis is still the Achilles heel of radical surgery for EC. The phase III trial NEOCRTEC5010 (25) shows that the overall recurrence rate after radical esophagectomy for locally advanced ESCC was 45% (159/353). Recurrence mainly occurred within 2 years after surgery (71.7%; 114/159).

How to overcome postoperative recurrence and metastasis has become a clinical challenge. In this context, TCM has great potential as a complementary therapy to current mainstream medicine. In previous studies of TCM interventions for EC, the outcome indicators primarily focused on short-term efficacy, such as quality of life and attenuation of adverse effects (26, 27). The participants were mostly in the middle to late stages of EC, and the effects of TCM were evaluated primarily when used as adjuvant therapy alongside radiotherapy or chemotherapy (28). A systematic review by Chen X et al. (11) included nine RCTs examining the clinical outcomes of TCM in treating patients with EC undergoing radiotherapy and chemotherapy. The results showed that the combined use of TCM positively influenced quality of life and increased patient tolerance to side effects caused by radiotherapy or chemotherapy. In terms of postoperative survival, a previous study on TCM focusing on OS and DFS in postoperative EC demonstrated that combining chemotherapy with TCM reduced the 3-year recurrence and metastasis rate by 23.3% and increased the 3-year survival rate by 14.1% compared with that of chemotherapy alone. It also significantly improved the quality of life and immune function of patients (29). However, a single-center, open-label design with incomplete randomization of the allocation sequence was employed in this study, which may not fully mitigate selectivity and implementation biases. Therefore, whether TCM alone improves the long-term prognosis of postoperative patients with EC still needs high-quality evidence to demonstrate.

This double-blind, multicenter, randomized, placebo-controlled clinical trial holds significant importance in investigating TCM as a complementary therapy to prevent recurrent metastasis after EC surgery. The advantages of this study are as follows: ①Central and block randomization was performed using the IWRS to ensure concealed allocation to balance unmeasured confounders. This approach maintained balanced non-experimental factors across multiple sub-centers competing for enrollment (30). ②Participants, care providers, investigators, and outcomes assessors were blinded to avoid placebo effect, information bias, and implementation bias, thereby obtaining more realistic trial data (31). ③The intervention timing was set at the end of postoperative adjuvant therapy, thereby eliminating the influence of confounding factors other than necessary symptomatic treatment on the outcome. ④The sub-centers of this study are primarily located in eastern China, where EC is highly prevalent, and the population is mainly Han Chinese (32). It is more typical and representative to evaluate the efficacy of QZYLP, while one limitation is that assessing other ethnic groups and regions will not be possible.

The main intervention measure of this study, QZYLP granules, was developed by Guang’anmen Hospital after extensive clinical practice and research. According to the theory of TCM, the occurrence and progression of EC are closely related to the pathological factors of phlegm and qi (33). Previous studies have shown that the obstruction of phlegm and qi are main factors affecting the recurrence and metastasis of EC after operation(*P*=0.019, *P*=0.016) (34). QZYLP is a targeted prescription of phlegm and qi in the postoperative patients with EC. It exhibits pharmacological action characterized by multi-element, multi-system, multi-target, and multi-mechanism effects. The primary components of QZYLP, such as *Astragali Radix* (35), *Codonopsis Radix* (36), and *Rhizoma Atractylodis Macrocephalae* (37), possess anticancer and immunomodulatory properties. Network pharmacology studies (38) showed that the potential targets of QZYLP interacted with multiple signaling pathways. One of the main roles was the regulation of oxidative stress. Phospholipids and cholesterol esters in cell membranes and lipoproteins readily reprogram lipid metabolism through the process of lipid peroxidation (39), which can alter the tumor microenvironment (TME) to promote tumor progression. Lipid accumulation impairs CD8^+^T-cell function in the TME and accelerates tumor growth (40), or enhances regulatory T-cell functional specialization in the TME (41). These processes eventually lead to immunosuppression and the progression of EC. In addition, our pre-metabolomics studies have found differences in the abundance of lipid metabolites in peripheral blood after QZYLP intervention. Therefore, we chose secondary outcome indicators related to lipid distribution and immunomodulation in order to elucidate the mechanism of action of QZYLP in biological terms.

To sum up, this study aims to further clarify the efficacy and safety of QZYLP in preventing postoperative recurrence and metastasis of EC, and to explore the mechanism of action. The results of this study will provide high-quality evidence for the participation of TCM in the comprehensive treatment program of EC, and improve the precise diagnosis and treatment system of TCM in EC. At the same time, the transformation of TCM will promote the clinical application, prolong the survival time, and enhance the benefit of patients in the future.

## 4 Trial status

The trial was prospectively registered at Clinical Trials. gov (ID: NCT05626309) on November 19,2022. Recruitment began in October 1,2022. Expected date when recruitment will be completed in August 30,2024. Due to COVID-19, the recruitment completion date will be extended.

## Data Availability

The original contributions presented in the study are included in the article/**Supplementary Material**. Further inquiries can be directed to the corresponding authors.
