# Supplementary material for: Qizhu Yuling prescription in the prevention of postoperative metastasis and recurrence of esophagus cancer: study protocol for a randomized, double-blind, placebo-controlled, multicenter clinical trial
Source: Front Oncol. 2025 Mar 26;15:1478390. doi: 10.3389/fonc.2025.1478390 (PMC11979610; doi:10.3389/fonc.2025.1478390)
Supplement: Supplementary file 1 [file Table1.docx]

Supplementary Material

# Supplementary Tables

**Attended Hospital**

| **Number** | **Name** |
| --- | --- |
| 1 | Guang 'anmen Hospital, China Academy of Chinese Medical Sciences |
| 2 | Beijing Hospital of Traditional Chinese Medicine |
| 3 | Xiyuan Hospital, China Academy of Chinese Medical Sciences |
| 4 | Wangjing Hospital, China Academy of Chinese Medical Sciences |
| 5 | Shandong Provincial Hospital of Traditional Chinese Medicine |
| 6 | Xingtai People's Hospital |
| 7 | Anyang Tumor Hospital |
| 8 | The Fourth Hospital of Hebei Medical University, Hebei Tumor Hospital |
| 9 | Chongqing University Cancer Hospital |
| 10 | Inner Mongolia Hospital of Traditional Chinese Medicine |
